# A community split among dolphins: the effect of social relationships on the membership of new communities

**DOI:** 10.1038/srep17266

**Published:** 2015-11-26

**Authors:** Miki Nishita, Miki Shirakihara, Masao Amano

**Affiliations:** 1 Graduate School of Fisheries and Environmental Science, Nagasaki University, 1-14 Bunkyo-machi, Nagasaki 852-8521, JAPAN; 2 Faculty of Science, Toho University, 2-2-1 Miyama, Funabashi, Chiba, 274-8510, JAPAN

## Abstract

Little is known about community splitting among dolphins because such events are rare in dolphin populations. A case of a community split was confirmed in a population of Indo-Pacific bottlenose dolphins (*Tursiops aduncus*) in Japan, where a group of approximately 30 dolphins moved to a new habitat some 60 km from the original habitat. We examined the associations among the dolphins before the community split to determine whether the new community members were already socially different before the split, using 7-year identification data. Before the split, the males in the same community after the split more often associated with each other than they did with those in different community. In contrast, the association patterns among females and between sexes showed no relationships with their post-split community membership. These results indicate that the males of new community were socially different from the other males for a long time before the split, but the females might not have been different. Our findings suggest that at time of the community split, the factors determining the memberships of the subsequent communities are sex-linked. The long-term social relationships among males could be maintained in the subsequent communities.

Many social mammals form cohesive and fairly closed social groups^1^. These groups occasionally split, and this phenomenon is called group fission, which refers to the permanent separation of members in a social group into two or more social groups^2^. The triggers of group fission may include increased group size, high cost of sexual competition among males, competition among females for resources and environmental stress, such a reduction in the available food^3^,^4^.

In social mammals that form fission-fusion societies, the group size and compositions of groups vary over periods of hours or days^5^. In such animals, a group representing a social unit is usually distinguished from the temporal group and defined by a different term, such as ‘unit-group’ or ‘community’ in chimpanzees (*Pan troglodytes*) and ‘clan’ in spotted hyenas (*Crocuta crocuta)*. It has been reported that these social units seldom split^6^,^7^. These splits of social units can be considered to be condition-dependent dispersal strategies and have consequences not only for the fitness of individuals but also for the population dynamics, genetics, and species’ distributions^8^. Therefore, understanding the causes and processes of splitting in these social units can be useful information for population management and the prediction of a population’s response to changes in the environment, the demographic traits of the population and human activities.

Bottlenose dolphins (*Tursiops* spp.) are known to form fission-fusion societies^9^. Despite these fluid associations, there are some patterns in associations among individuals^10^,^11^,^12^. For example, female dolphins have been found to associate with other females in a similar reproductive condition^13^, and pairs or trios of males form strong, long-term associations, called alliances^14^,^15^,^16^,^17^. It is thought that females with similar reproductive conditions share similar requirements for food and protection from predators or from harassment by males, whereas alliance relationships between males facilitate access to females in estrous^16^. Therefore, these relationships function as important social strategies for these dolphin populations^18^.

Some coastal bottlenose dolphin populations are divided into sub-populations, called communities, and these are recognized by shared patterns of residency and associations among individuals^19^. Many studies have focused on identifying the community structure within a population of coastal dolphins^19^,^20^,^21^,^22^,^23^, and such information enables researchers to define the appropriate management units^24^.

However, few studies have documented the splitting of a community or population, probably because such events are very rare. Elliser and Herzing have documented a split in a population of resident bottlenose dolphins (*T. truncatus*) off Grand Bahama Island, which divided into two distinct units^25^. The authors suggest that the loss of many individuals within the population as a result of hurricanes in the area may have disrupted the communication between individuals and facilitated the community splitting. To our knowledge, this is the only published occurrence of a split in a cetacean community.

The population of Indo-Pacific bottlenose dolphins (*T. aduncus*) around Amakusa-Shimoshima Island in western Kyushu, Japan is currently composed of two communities^26^ (Fig. 1). The first is the northern community (ca. 200 individuals), located off the northern waters of Amakusa-Shimoshima Island. The northern community members form large fission-fusion groups of approximately 80 individuals, and sometimes exceed 100 individuals^27^. The other is the southern community (ca. 20 individuals) and their group structure is stable. The dolphins of both communities originally inhabited the northern waters of Amakusa-Shimoshima Island, which is now the current range of the northern community. However, in 2000, many shifted their habitat to the southern waters of the island, approximately 60 km from the original habitat. From March to May in 2001, many returned to the north, whereas some dolphins remained and established the southern community.

Factors such as increased community size, decreased food availability and an excessively biased sex ratio in a community could be the causes of community splitting, but in this case, the causes of the split are unknown. Anthropogenic factors, such as the dolphin-watching industry, might cause stress among some members of the original population. Dolphin-watching tours have been run in the study area since 1993, and changes in dolphin behavior in response to the tour boats have been documented^28^.

Although it is not possible to ascertain what caused the split, the available data on the community structure both before and after the split provided us with a valuable opportunity to investigate the effect of the existing social relationships within and between sexes on the composition of new communities. Wiszniewski *et al.* have proposed that segregation of communities results from individuals’ adaptation to local environmental conditions, facilitated by the variability in the individuals’ association preferences^29^. Lusseau and Newman have demonstrated that community separation probably arose from the preferential assortment of individuals by sex and age class, using network analyses^30^. Although these studies were mere conjectures based on the already existing community structure and have never verified the community splitting, the individuals’ association preferences may play a role in the process of establishing a community.

We examined the existence and strength of the social relationships among the Amakusa-Shimoshima bottlenose dolphins before the split to assess whether the existing relationships defined the composition of the two communities that resulted from the splitting of this population. The findings provide some insight into the rarely documented occurrence of a split in a population of dolphins and the factors that may determine the structure of the cetacean communities after such an event.

## Results

A total of 123 sexed individuals were identified on more than 6 days for at least 4 years of the 7-year study period, including 54 males that inhabited the northern coast of the island after the split (hereafter NC males), 14 males that inhabited the southern coast of the island after the split (hereafter SC males), 51 females that inhabited the northern coast of the island after the split (hereafter NC females) and 4 females that inhabited the southern coast of the island after the split (hereafter SC females).

### Variances in associations

To quantify the strength of the relationship between individuals, we calculated the half-weight association index (HWI) and the half-weight association index gregariousness (HWIG), which corrects the association index between dyads for their respective levels of gregariousness^31^. The reason for using HWIG is that animal populations can show variation in individual gregariousness, with some individuals being found mainly in small groups or having few associates, and others generally being found in larger groups or having many associates^27^,^32^.

The correlation of the variation of the true association indices was higher than that of the random association indices estimated by random permutations of the actual data, using HWI and HWIG as the association indices in both cases (P = 0.0087 and P = 0.0082, respectively). This indicates that the individuals did not randomly associate with others.

### Differences in the association preferences between the NC and SC members

To determine whether the associations between individuals before the split were based on the post-split community memberships, we classified the individuals into two community classes (NC and SC) based on their habitat after the split and compared the association preferences within and between community classes. Figure 2 shows the comparisons between the association indices (HWI and HWIG) among individuals within the community class (NC-NC and SC-SC) vs. those between the community classes (NC-SC) for both males and females.

#### Associations among males

The association indices among males within the community class were higher than those between the community classes in both cases, using the HWIs and HWIGs as association indices (Mantel test, P < 0.00001, respectively). This result indicates that the males in the same community class were more likely to associate with each other than they were with those in the other community class. To examine the details of the association relationships among males, a dendrogram was constructed using average-linkage hierarchical cluster analysis for males, as shown in Fig. 3 33. In this dendrogram, most of the males in the same community class were clustered together, with the exceptions of NC male #220 when we used the HWI as the association index, and SC males #142, #94 when we used the HWIG as the association index (Fig. 3). In the clustering using the HWIG as the association index, the SC males were more tightly clustered than the NC males (Fig. 3). We compared the association indices among the NC males and those among SC males to determine whether the SC males exhibited stronger associations than the NC males (Fig. 4). The randomization test revealed that the HWIs among NC males were significantly higher than those among SC males (P < 0.0001). In contrast, the HWIGs among the SC males were significantly higher than those among the NC males (randomization test, P < 0.0001). The higher HWIs among the NC males than the SC males indicated higher gregariousness among the NC males than the SC males. Given their differences in gregariousness, the associations among SC males were stronger than those among the NC males.

#### Associations among females

We compared the association indices using both the HWIs and HWIGs among females within the community class (NC-NC and SC-SC) vs. those between the community classes (NC-SC) to determine whether the females’ associations before the split were based on the post-split community memberships. There were no significant differences between the association indices (HWI and HWIG) among females within the community class and between the community classes (Mantel test, P = 0.24475 and P = 0.6705, respectively, Fig. 2).

#### Associations between sexes

We compared the association indices of the NC males-NC females vs. those of NC males-SC females, as well as those of the SC males-NC females vs. those of SC males-SC females to examine whether the males tended to associate more often with females of the same community class as they did before the split. As shown in Fig. 5a, there were no differences in either the HWIs or HWIGs between the community classes of females for both the NC and SC males, indicating both the NC and SC males associated with the females, regardless of the females’ community class. Although this result showed that the males did not exhibit a preference for the females on the basis of post-split community memberships, the association indices of the NC males-all females were significantly greater than those of the SC males-all females in both the HWIs and HWIGs, indicating that the NC males associated with females more often than the SC males (randomization test, P = 0.0001, respectively, Fig. 5b).

### Temporality in the associations among male dolphins

To examine the temporal patterns of the associations, we calculated the standardized lagged association rate (which is “an estimate of the probability that if two individuals are associated at any time, then, after the specified lag, the second individual is a randomly chosen associate of the first”) for the NC and SC males and fitted four models to the data^34^,^35^. These four models and their quasi Akaike Information Criterion (QAIC) values are shown in Table 1 and the best fitting models and supported models are shown in Fig. 6. Based on the lowest QAIC value, the “casual acquaintances” model was chosen for NC males, and the duration of the associations was estimated to be on the order of several decades. The “preferred companions” model was chosen as the best-fitting model for SC males, with minimum QAIC value. However, the “casual acquaintances” and “constant companions and casual acquaintance” models, whose ΔQAIC were 1.7051 and 1.1699, respectively, were also strongly supported models, because the models with ΔQAIC = 0–2 are considered to be strongly supported models in addition to the best fit model (Table 1). As shown in Fig. 6, the graphs of best two models for the SC males (“preferred companions” and “constant companions and casual acquaintances”) were very close to each other and there was no or very little slope in the graph, indicating that the association relationships among SC males rarely changed with time.

### Reproductive conditions of SC females at the community split

It has been reported that female dolphins associate with other females based on their reproductive conditions^13^. To determine whether the relationships among females based on reproductive conditions affected the composition of new community, we confirmed the females’ reproductive conditions based on the observation of their offspring though the identification survey in the southern part of the island in May 2001, when the community split occurred. Table 2 shows the reproductive conditions of the SC females at this time. This table showed that the reproductive conditions of SC females varied.

## Discussion

Most of the males in each community class, which were classified based on the post-split community memberships, were clustered together, and the males in the same community class were more likely to associate with each other than they were with those in the other community class before the community split occurred. These results indicate that the males in each community were already socially different before the community split. As indicated by the result of the model fitting for the standardized lagged association rate, the best fitting model for the NC males was “casual acquaintances” and the estimated duration of the associations from this fitted model was on the order of several decades. This result indicated that the associations among NC males were not necessarily stable but lasted for long time. The models fitted for the SC males indicated that association relationships among SC males rarely changed with time, which indicates that the social relationships among SC males were very stable. In addition, the HWIGs among SC males were higher than those among NC males, while the HWIs among SC males were not higher. These contrasting results indicate that the SC males were less gregarious, but their associations with each other were strong. Thus, these results suggest that the SC males had strong social bonds because of their gregariousness and their long-lasting relationships. It is known that male dolphins form long-term relationships, termed alliances, and the relationships among alliance members can last for several years, up to 17 years^14^,^15^,^16^,^17^. Males in alliances are thought to cooperate to obtain access to receptive females^16^,^36^. The SC males in this study population may have formed alliances, but the data collected are not sufficient to demonstrate the presence of such alliance relationships among males because we have no behavioral data on the interactions between males and females, such as the males’ consortships with females^14^,^15^,^16^,^17^. The long-term relationships lasting for at least seven years, which may or may not be alliances, might have been an underlying factor in the eventual composition of the male dolphins in the split communities.

There were no differences between the association indices among females within the community class vs. those between the community classes in the 7-year pooled data. This indicates that prior to the split, the females of both community classes associated with each other, regardless of the post-split community memberships. It is known that female bottlenose dolphins usually show stable, moderate level associations with other females within social clusters, named “bands” or “cliques”^10^,^12^,^20^,^37^. If the social relationships among females were linked to the community membership of the females after the split, the females in the two communities should have associated more often before the split, but this was probably not the case. Female bottlenose dolphins tend to associate based on the reproductive conditions within social clusters^13^. Connor *et al.* have proposed that female bottlenose dolphins with similar reproductive conditions might benefit from associating with each other because they share similar requirements for food and protection^9^. However, the reproductive conditions of SC females were not similar at the time of the community split, and those females’ associations did not appear to be based on shared reproductive conditions. Therefore, neither long-term social relationships nor relationships based on reproductive conditions among females affected the structure of the new communities in this study.

The results of the comparisons of the association indices between sexes in each community class indicated that the males associated with females, regardless of the females’ community classes. Furthermore, both the NC and SC females associated with the NC males more than the SC males before the community split. These results imply that there was little effect of the long-term social relationships between sexes on the memberships of the new community in this study. In group fission in primates, special associations between particular males and females can trigger or promote group fission^4^,^38^,^39^. Based on their anecdotal observations, Tsai and Mann have suggested that the behavior of male bottlenose dolphins (*Tursiops* sp.) affects females’ habitat use and range, because male alliances herded females from adjacent areas into the their main study area; these events sometimes lasted for several weeks^40^. Although we were not able to investigate the short-term relationships near the community split because of insufficient data, particularly in 1999 and 2000, it might have been possible that the SC males increased their associations with SC females just before the community split. Moreover, the SC males’ behavior may have affected the SC females’ habitat use and range, even if the SC males associated with the females less often throughout the 7-year study period. In fact, four of the seven SC females did not have a calf or juvenile when the community split. These females without calves might have been particularly attractive to males and the SC males may have exhibited herding-like behavior when the community split.

When splits in mammalian social groups occur, social relationships, including dominance relationships and kinship, can influence the memberships of the new groups (e.g., in the Japanese macaque, *Macaca fuscata*^38^ and olive baboon, *Papio cynocephalus*^39^). In spotted hyenas, dominance rank influences the memberships of new clans, which are structured by stable linear rank relationships^41^. However, in moor macaques (*M. maurus*), which do not have a hierarchical community structure among females, neither kinship nor association patterns established before the group fission strongly affect the memberships of the new groups^42^. The difference in the female reproductive conditions between the two branch groups was evident and was linked to the structure of the new group. The authors also suggest that the effect of the reproductive conditions on the furcation patterns of females may be expressed more clearly in egalitarian species, in which social interactions are less influenced by dominance or kinship^42^. In contrast, in the community split among female dolphins in this study population, the effect of reproductive conditions on the membership of the new community was not apparent; however, that does not necessarily mean that the dolphins in this study population are not egalitarian and dominance rank and kinship have a greater effect on the splitting pattern among females. The dominance hierarchies among dolphins have been confirmed only in captive bottlenose dolphins^43^, and we do not have access to information on dominance relationships and kinship in this study population. Thus, the effect of dominance rank and kinship cannot be evaluated. Future work should focus on the genetic and dominance structures.

In conclusion, this study resulted from a unique opportunity to examine the effect of social relationships on the process of community splitting. Our results imply that the factors determining the community structure after a community split are sex-linked, and the males’ long-lasting relationships play an important role in the post-split structure of the communities.

## Methods

### Data collection for social analyses

**Figure 1:**
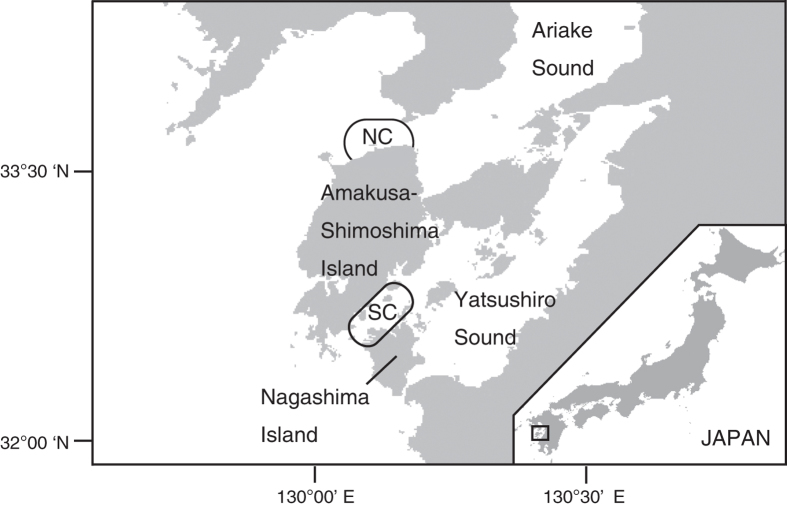
Study area around Amakusa-Shimoshima Island in western Kyusyu, Japan. NC shows the range considered to be the stable daytime habitat for the northern community (Ken Inoue, pers. comm.). SC is the area where the southern community dolphins were observed during the daytime. Most of the photo-identification surveys were conducted in the NC area. The maps were created by using ArcGIS.

**Figure 2:**
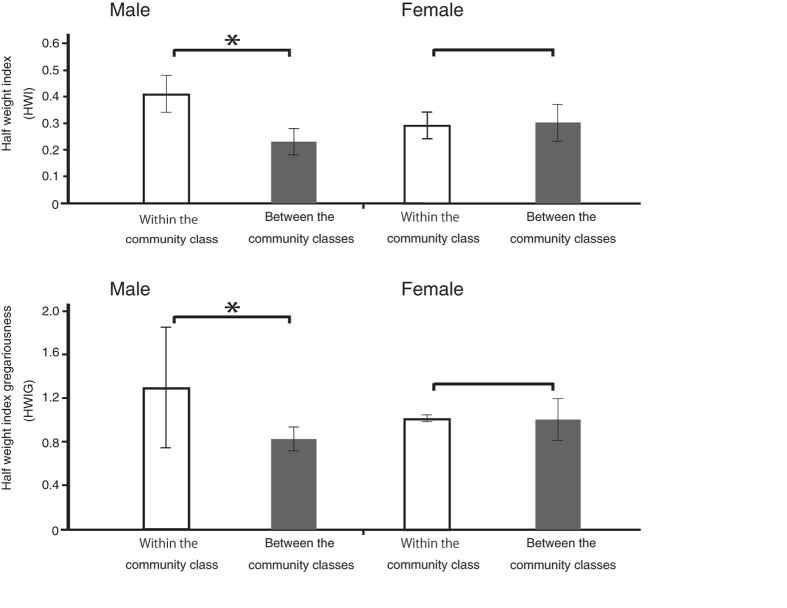
Comparisons between the association indices among individuals within the community class vs. those between the community classes for each sex, using both the HWI and HWIG values. The individuals were classified into two community classes based on their post-split community memberships. The white bars show the mean values of the association indices among the individuals in the same community class (NC-NC and SC-SC) and the black bars show those of the individuals between the community classes (NC-SC) for each sex. The error bars represent the standard deviations. * indicates significance at P < 0.05 (Mantel test).

**Figure 3:**
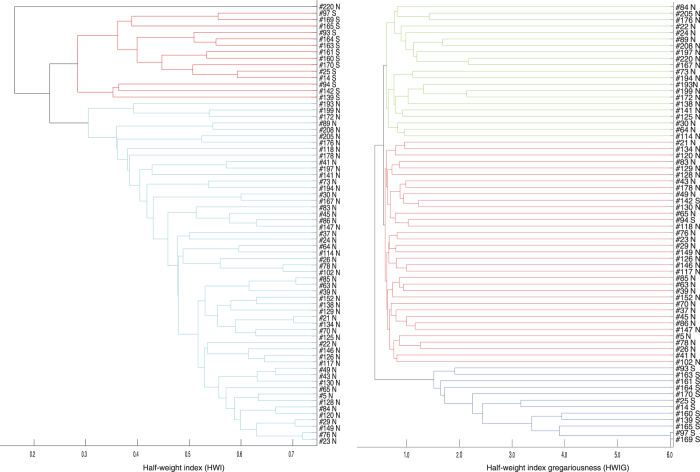
Dendrogram constructed from the average-linkage hierarchical cluster analysis for male dolphins between 1994–2000, using the HWI and HWIG values. The individuals are represented by an ID number with the letter N or S, each of which indicates the community class. The cophenetic correlation coefficient for the clustering using HWI is 0.82 and that using HWIG is 0.80. The cophenetic correlation coefficient values greater than approximately 0.8 indicate a good match of the dendrogram to the association matrix.

**Figure 4:**
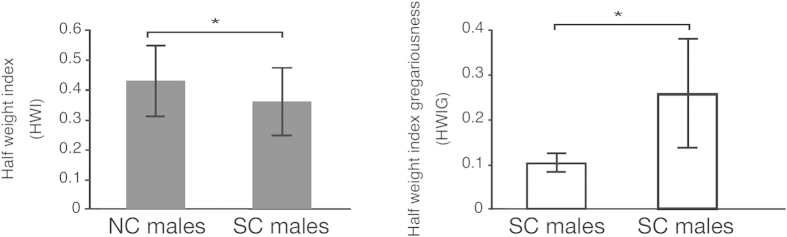
Comparisons between the association indices (HWI and HWIG) among NC males and those among SC males. The error bars represent the standard deviations. * indicates significance at P < 0.05 (Mantel test).

**Figure 5:**
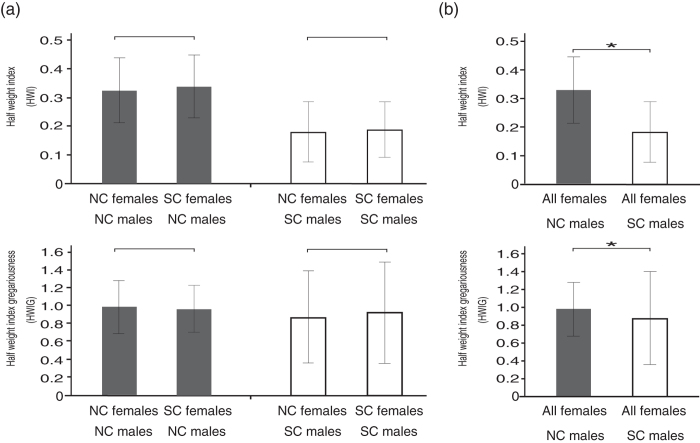
Comparisons of the association indices (both HWI and HWIG) between sexes for each community class. (**a**) NC males-NC females vs. NC males-SC females (black bars) and SC males-NC females vs. SC males-SC females (white bars). (**b**) NC males-all females vs. SC males-all females. The individuals are classified into two community classes based on their post-split community memberships. The error bars represent the standard deviations. * indicates significance at P < 0.05 (Randomization test).

**Figure 6:**
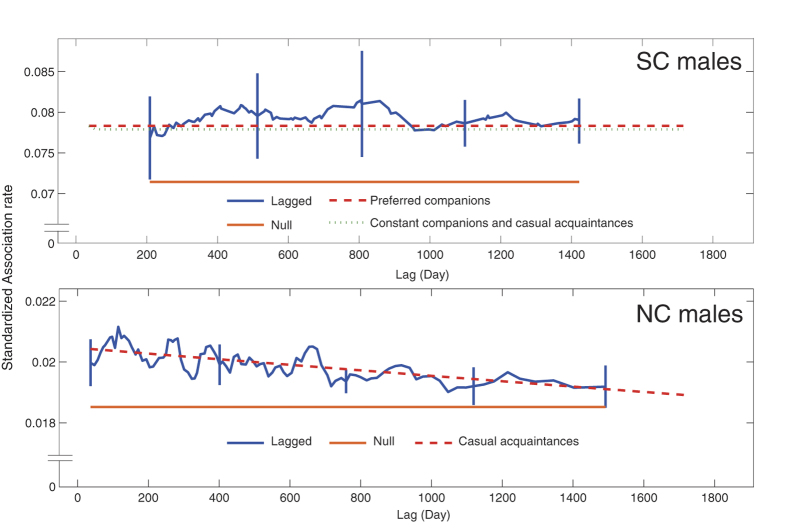
Standardized lagged association rates (SLAR) for the NC and SC males, with jack-knifed estimates of precision. The null associations (the theoretical SLAR if the individuals are randomly associated) and the best fitting models are shown for the NC and SC males. The supported models for the SC males are also shown.

We used the photo-identification data from 114 surveys conducted off the northern coast of Amakusa-Shimoshima Island, in western Kyushu, Japan on 84 days between 1994 and 2000, before the individuals moved to the southern region, for the social analyses (Fig. 1). We approached a large group of dolphins, which, in most cases, we had spotted from the land in advance, and we photographed the dorsal fins, using a 35-mm camera (CANON EOS) with a 75–300-mm zoom lens and color slide film (FUJICHROME, ASA100–400). Each survey was 1–3 hours in duration and one to three surveys were performed each day by boat (5–10 m in length). The individuals identified on more than 6 days over at least 4 years were used for the analyses. All procedures adhered to the Japanese “Act on Welfare and Management of Animals”, the Code of Conduct issued by Amakusa dolphins-watching industry, and the Guideline for Ethological Studies issued by the Japan Ethological Society.

### Definition

The individuals were divided into two categories based on the present communities; the individuals inhabiting the northern community area after the community split were defined as the NC members, whereas those inhabiting the southern community area after the split were defined as the SC members. The sex of an individual was determined based on the presence of calves in photo-identification data between 1994 and 2013^44^. We regarded an individual as a female when it was repeatedly observed with a small animal, presumed to be her calf, on different days and for multiple seasons to establish reliable mother-calf relationships. Three reproductive condition categories at the time of the community split were distinguished for SC females based on the presence of a dependent calf or juveniles: a female with a calf (<1 yr), a female with a juvenile (>1 yr), or a female without calves or juveniles. Individuals that had been identified for 10 years or more and had never been observed with a calf during that time were considered as males. Dolphins that already had distinctive notches on their dorsal fins, were independent from their mother when first encountered and identified, and that had never been observed with a calf or juvenile for at least 9 years of observation were also categorized as males. The period of 9 years was chosen because the inter-birth intervals are shorter than 8 years in this species^45^. The individuals for which we were unable to determine the sex were excluded from the analyses.

### Social analyses

The dolphins in this study area usually form a large group of approximately 80 individuals or more. Thus, in most surveys, this group was approached to collect photo-identification data^27^. For this reason, the dolphins’ associations were defined as all individuals sighted during a survey. The sampling period was set as one day to avoid the replication of associations within the same day. To quantify the strength of the relationships between individuals, we used the half-weight index (HWI) and the half-weight index gregariousness (HWIG), a version of the HWI that controls for individual variations in gregariousness, as calculated by following formula^31^:


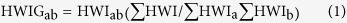


where HWIG_ab_ is the HWIG between individual a and individual b, HWI_ab_ is the HWI between individual a and individual b, ∑HWI is sum of the HWI values of all individuals, ∑HWI_a_ is sum of the HWI values of individual a, and ∑HWI_b_ is the sum of the HWI of individual b. Whereas an HWI equal to zero indicates that the dyad never associated and an HWI equal to one indicates that the dyads were always observed together, an HWIG lower than one indicates that the dyad was associated less often than expected giving the dolphins’ gregariousness, and an HWIG higher than one indicates that the dyads associated more often than expected given their gregariousness^31^. The permutation test was conducted to test the nonrandom associations among individuals^46^. In this study, ‘permutate groups within samples’ test was used and the association matrix was randomized 10,000 times with 100 flips per permutation, at which point the P-values stabilized. A significantly higher value of the coefficient of variation (CV) of the observed association indices compared to the randomly permutated association indices indicates nonrandom associations among individuals. A Mantel test was performed for each sex to compare the mean value between the HWIGs among the individuals within a community class vs. those between the community classes. The average linkage cluster analysis was conducted for both sexes, but we only showed the results of the cluster analysis for the males because we were unable to obtain enough cophenetic correlation ratio values for the females. To examine the temporal patterns of associations, the standardized lagged association rates (SLAR) were calculated for the males in each community, and four models were fitted to the SLAR to describe the males’ temporal association patterns^34^,^35^. These models included “preferred companions”, where the rate is constant and represents permanent associations; “casual acquaintances”, where the rate decays down to zero and represents the associations that occurred for a given time lag and then never again; “constant companions and casual acquaintances”, where the rate decays down to a lower level after a given time lag and then levels off and represents an association followed by disassociation at some time lag to a lower level of association at which the associations stabilize; and “two levels of casual acquaintances”, which is the sum of two exponential delay processes down to zero and represents an association and disassociation occurring on two different time scales. Jackknifing techniques were used to calculate the standard error. The best fitting model was chosen based on lowest quasi Akaike Information Criterion (QAIC) and the ΔQAIC (difference between QAIC and that of the best model) was used to estimate how each model was supportive, other than the best fitting model^47^. The randomization test was conducted to compare the means of HWIGs between males and females for each community with 10,000 randomizations, using the RT-Heritage programs^48^.

Unless otherwise noted, all social analyses were conducted with SOCPROG 2.5^49^.

## Additional Information

**How to cite this article**: Nishita, M. *et al.* A community split among dolphins: the effect of social relationships on the membership of new communities. *Sci. Rep.* **5**, 17266; doi: 10.1038/srep17266 (2015).

## Figures and Tables

**Table 1:** Models for the standardized lagged association rate for the males of each community class.

Community class	Model’s explanation	Fitted model (association rate)	QAIC	ΔQAIC
NC males	Preferred companions	0.019949	668986.0494	20.2994
	Casual acquaintances	0.020463*exp(−4.5892e-05*td)	668965.7500	0.0000
	Constant companions and casual acquaintances	0.019945 + 0.0020907*exp(−1.3062*td)	668989.7059	23.9559
	Two levels of casual acquaintance	0.020463*exp(−4.5895e-05*td) + 8.6092e-05*exp(−1.0382*td)	668969.7490	3.9990
SC males	Preferred companions	0.07832	14069.5029	0
	Casual acquaintances	0.077113*exp(−1.9482e-05*td)	14071.208	1.7051
	Constant companions and casual acquaintances	0.077888 + 0.050977*exp(−0.13126*td)	14070.6728	1.1699
	Two levels of casual acquaintances	30.1169*exp(−32.8988*td) + 0.077096*exp(−1.9541e-05*td)	14075.2079	5.705

The lowest quasi Akaike Information Criterion value (QAIC) indicates the best fitted model for the males of each community. ΔQAIC is the difference between the QAIC value for the current model and that of the best model.

**Table 2:** Reproductive conditions of the SC females in May 2001, after the community split.

ID	Reproductive conditions
#28	Without calves or juveniles
#145	Without calves or juveniles
#158	Without calves or juveniles
#91	With a juvenile (>1yr old)
#201	With a juvenile (>1yr old)
#68	With a calf
#5000	Without calves or juveniles but assumed to be pregnant because she was observed with a calf in August 2001.
